# Near-Infrared Light Driven ZnIn_2_S_4_-Based Photocatalysts for Environmental and Energy Applications: Progress and Perspectives

**DOI:** 10.3390/molecules28052142

**Published:** 2023-02-24

**Authors:** Yi Cai, Fangxin Luo, Yujun Guo, Feng Guo, Weilong Shi, Shengtao Yang

**Affiliations:** 1 Key Laboratory of Pollution Control Chemistry and Environmental Functional Materials for Qinghai-Tibet Plateau of the National Ethnic Affairs Commission, School of Chemistry and Environment, Southwest Minzu University, Chengdu 610041, China; 2 School of Energy and Power, Jiangsu University of Science and Technology, Zhenjiang 212003, China; 3 School of Material Science and Engineering, Jiangsu University of Science and Technology, Zhenjiang 212003, China

**Keywords:** ZnIn_2_S_4_, photocatalysis, hydrogen evolution, near-infrared light

## Abstract

Zinc indium sulfide (ZnIn_2_S_4_), as a significant visible-light-responsive photocatalyst, has become a research hotspot to tackle energy demand and environmental issues owing to its excellent properties of high stability, easy fabrication, and remarkable catalytic activity. However, its drawbacks, including low utilization of solar light and fast photoinduced charge carriers, limit its applications. Promoting the response for near-infrared (NIR) light (~52% solar light) of ZnIn_2_S_4_-based photocatalysts is the primary challenge to overcome. In this review, various modulation strategies of ZnIn_2_S_4_ have been described, which include hybrid with narrow optical gap materials, bandgap engineering, up-conversion materials, and surface plasmon materials for enhanced NIR photocatalytic performance in the applications of hydrogen evolution, pollutants purification, and CO_2_ reduction. In addition, the synthesis methods and mechanisms of NIR light-driven ZnIn_2_S_4_-based photocatalysts are summarized. Finally, this review presents perspectives for future development of efficient NIR photon conversion of ZnIn_2_S_4_-based photocatalysts.

## 1. Introduction

Photocatalytic technology is recognized as a potential eco-friendly approach to address the issues of environmental pollution and energy shortage by utilizing solar light. A host of photocatalysts have been synthesized for efficient photocatalytic activity, e.g., TiO_2_ [1,2], metal oxides [3], metal nitrides/sulfides [4,5,6,7], graphitic carbon nitride (g-C_3_N_4_) [8,9,10], metal–organic frameworks (MOF) [11,12], and covalent organic frameworks (COF) [13,14]. Recently, zinc indium sulfide (ZnIn_2_S_4_), as a member of the ternary AB_2_X_4_ family, aroused researchers’ interest in the photocatalysis field [15,16]. It exhibits excellent properties of visible light absorption owing to its suitable band gap (~2.5 eV), which leads to abundant generation of photoinduced carriers under visible light irradiation [17,18,19]. Additionally, it shows remarkable chemical stability, sharing similar characteristics with other metal sulfides [20,21]. Moreover, it has the properties of stable and easy fabrication, which makes it feasible for practical applications. Benefitting from these advantages, the applications of ZnIn_2_S_4_ have been reported for water splitting [22,23,24,25,26,27,28,29,30,31], CO_2_ reduction [32,33], Cr(VI) reduction [34,35,36], nitrogen reduction, [37,38,39] pollutant removal [40,41,42,43], etc.

Although ZnIn_2_S_4_ exhibits excellent photocatalytic activity in the UV–visible light range, it has poor photocatalytic activity in near-infrared light (NIR) [44,45] due to its low response towards NIR. The solar spectrum consists of ultraviolet (UV, 300–400 nm), visible (400–700 nm), and near-infrared (NIR, 700–2500 nm) regions, which account for of 5%, 43%, and 52%, respectively [44,45,46]. Therefore, the NIR light absorption of ZnIn_2_S_4_-based should be extended to improve solar light utilization, and the fabrication of NIR-responsive ZnIn_2_S_4_ photocatalysts is the crucial approach to promote its photocatalytic efficiency for further practical applications. There are three mechanisms to promote utilization of near-infrared light by ZnIn_2_S_4_: (i) constructing narrow bandgap semiconductors such as Ag_2_S [47], Ag_2_O [48], and Bi_2_WO_6_ [49]_,_ etc. to absorb NIR light directly to generate electron hole carriers for photocatalytic redox reactions; (ii) coupling with up-conversion materials such as carbon quantum dots (CQDs), Cu_2_(OH)PO_4_, etc., to convert NIR light to high-energy UV–visible light, thus promoting the NIR photocatalytic activity; and (iii) the photothermal effect, in which photothermal composites convert NIR light to thermal energy and boost photocatalytic activity [27,33,50,51,52]. In order to extend the NIR light absorption range and sperate photoinduced charge carriers, several strategies have been applied to fabricate ZnIn_2_S_4_-based NIR light-driven photocatalysts, including bandgap engineering, construction of narrow optical gap materials, up-conversion, and plasmonic materials [53,54,55,56].

Herein, recent progress in NIR-driven ZnIn_2_S_4_ photocatalysts for the application of hydrogen evolution, pollutant removal, and CO_2_ reduction is overviewed. Firstly, different construction strategies of ZnIn_2_S_4_-based photocatalysts to allow full utilization of NIR in the solar spectrum are comprehensively summarized, including hybrid with narrow optical gap materials, bandgap engineering, up-conversion materials, and plasmonic materials. Focus then turns to the applications of photocatalytic water splitting, organic pollutants removal and CO_2_ reduction. Recent research into strategies and applications of NIR-driven ZnIn_2_S_4_-based photocatalysis is summarized in the table below (Table 1). In addition, this review discusses the mechanisms of ZnIn_2_S_4_-based NIR light-driven photocatalysts in the respective photocatalytic systems. Finally, current challenges and perspectives for the future development of efficient NIR-responsive ZnIn_2_S_4_-based photocatalysts are put forward.

## 2. Hybrid with Narrow Optical Gap Materials

Narrow optical gap materials (NOGMs, e.g., Ag_2_O, CuInS_2_ and black phosphorus) have an inherent response to visible–NIR light. However, the rapid recombination of photo-induced carriers and band positions limits their application in photocatalysis. Therefore, the use of traditional narrow bandgap semiconductors by itself is inefficient and impractical for photocatalytic utilization. Constructing binary or ternary heterostructures photocatalysts consequently served as a potential candidate for achieving distinguished photocatalytic performance. In recent years, more attention has focused on coupling ZnIn_2_S_4_ with NOGMs for NIR light-driven photocatalysis as a result of enhanced NIR-driven photoactivity and photo-generated charge carriers.

**Figure 1:**
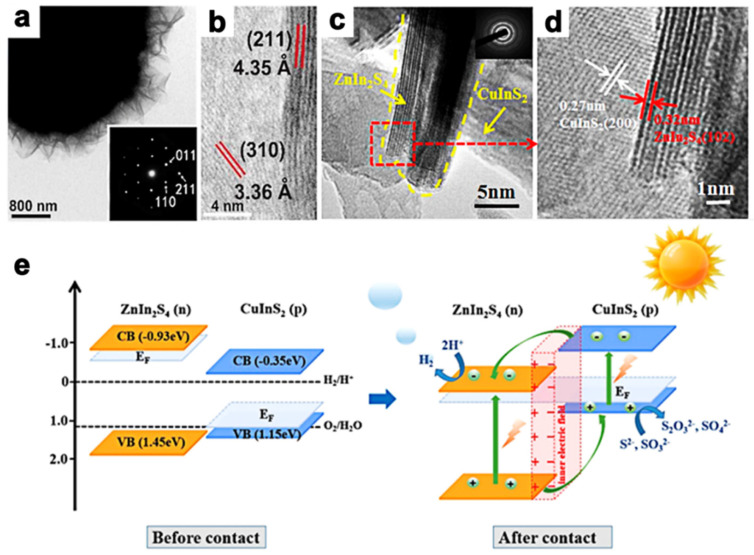
TEM (**a**) and HRTEM (**b**) of AgIn_5_S_8_/ZnIn_2_S_4_ heteromicrospheres. Reprinted with permission from Ref. [57]. Copyright 2015, Royal Society of Chemistry. (**c**,**d**) HRTEM of ZnIn_2_S_4_@CuInS_2_ composites [53]. (**e**) Schematic diagrams of the formation of *p–n* junction and proposed charge separation process in the ZnIn_2_S_4_@CuInS_2_ core–shell photocatalysts. Reprinted with permission from Ref. [53]. Copyright 2020, American Chemical Society.

Metal chalcogenides exhibit a broad-band absorption property in the visible to NIR range with a narrow bandgap. For instance, Xu et al. [57] fabricated AgIn_5_S_8_/ZnIn_2_S_4_ heteromicrospheres with visible–NIR absorption characteristics in the range of 200–810 nm synthesized by a partial cation exchange method. TEM (transmission electron microscopy, (Figure 1a)) and HRTEM (high-resolution transmission electron microscopy, (Figure 1b)) show that AgIn_5_S_8_ flake-like structures were distributed on the surface of ZnIn_2_S_4_, proving the formation of a heterojunction. The absorption edge of heterostructure shows the redshift from 593 nm to 810 nm compared to pristine ZnIn_2_S_4_, and the Rhodamine B (RhB) photodegradation rate reached 99.2% within 50 min. Additionally, the thickness of AgIn_5_S_8_ could be a crucial factor affecting the photodegradation efficiency from the joint results of photodegradation and X-ray photoelectron spectroscopy (XPS) analysis [57]. In another study, Yu et al. [53] prepared ZnIn_2_S_4_@CuInS_2_ microflowers through a two-step hydrothermal approach, and HRTEM images (Figure 1c,d) displayed that CuInS_2_ wrapped on the surface of ZnIn_2_S_4_, which indicated the successful integration between CuInS_2_ and ZnIn_2_S_4_. The absorption region was extended from visible light to the NIR region owing to the good construction of CuInS_2_ and ZnIn_2_S_4_. Moreover, further characteristics such as the photocurrent and chemical impedance spectroscopy, etc., led to the generation of more photoinduced electrons and a higher performance of charge transfer. Consequently, the ZnIn_2_S_4_ integrated with 0.05% CuInS_2_ exhibited a remarkable hydrogen evolution rate of 1168 μmol g^−1^ under λ > 420 nm light irradiation. The mechanism (Figure 1e) demonstrated that the remarkable photocatalytic activity was contributed to by the built-in electric field between ZnIn_2_S_4_ and CuInS_2_, which created a larger interface contact area and thus promoted charge separation and transfer. Moreover, a slight decrease in hydrogen evolution photoactivity was observed in the recycling reactions due to the photocorrosion effect of the hole consumption [64,65] and stability of the ZnIn_2_S_4_@CuInS_2_ materials was confirmed by SEM and TEM carried out before and after the reactions.

Research on hybrid with narrow optical gap materials is in its early stages, and combined strategies should be considered to balance the benefits of broad solar light absorption by NOGMs due to its narrow bandgap, and its drawbacks of fast photoinduced carrier recombination. Moreover, the innovative design of ZnIn_2_S_4_-based photocatalysts could avoid their photocorrosion and promote their recycling and stability performance.

## 3. Bandgap Engineering

**Figure 2:**
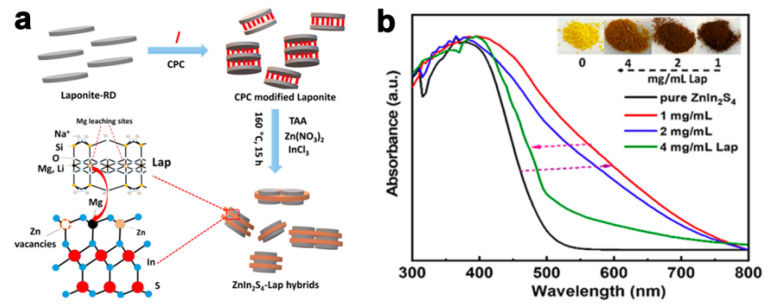
(**a**) Schematic illustration of ZnIn_2_S_4_–Laponite synthesis, where CPC and Lap represent cetylpyridinium chloride and laponite, respectively. (**b**) UV–vis diffuse reflectance spectra of inset samples with different amounts of Laponite at 1, 2, and 4 mg mL^−1^, respectively. Reprinted with permission from Ref. [54]. Copyright 2021, American Chemical Society.

In addition to incorporating NIR-spectrum response semiconductors, bandgap engineering (BGE) is a promising strategy for fabricating NIR-responsive ZnIn_2_S_4_. The introduction of atom doping, defects, and disorders is a common approach applied to modify the ZnIn_2_S_4_ electronic structure and composition stoichiometry and thus enhance the light utilization to the NIR range. However, only a few cases of bandgap engineering of ZnIn_2_S_4_ have been reported for construction of NIR-responsive photocatalysts. For instance, Hill et al. reported the formation of Zn-defective ZnIn_2_S_4_–Laponite heterostructures due to Mg^2+^ leached from Laponite in the synthesis process (Figure 2a); leached Mg^2+^ replaced the Zn in ZnIn_2_S_4_ owing to their similar ionic radii, which led to the formation of Zn defects in ZnIn_2_S_4_. Due to this engineering defect, the absorption range of the ZnIn_2_S_4_–Laponite samples was broadened from UV to NIR at approximately 800 nm (Figure 2b). Furthermore, higher charge separation efficiency and greater photoinduced charge were observed by using transient photocurrent measurements and electrochemical impedance spectroscopy (EIS) methods. Eventually, the photocatalytic degradation of methyl orange (MO) by ZnIn_2_S_4_–Laponite was 3.3 times more efficient than pure ZnIn_2_S_4_, which could be attributed to the formation of Zn vacancies in ZnIn_2_S_4_ acting as electron capture centers and therefore promoting the photoinduced charge separation [54].

To date, beyond introducing defects, other bandgap engineering methods such as atom doping and disorders, etc., on ZIS have not yet been explored. These bandgap engineering approaches could be feasible ways to enhance the absorption of NIR light and therefore promote photocatalytic efficiency.

## 4. Up-Conversion Materials

Up-conversion is an anti-Stokes luminescence process that derives from sensitized triplet–triplet annihilation, converting low-energy pump photons to higher-energy emission (UV or visible light photons). The up-conversion process makes it feasible to exploit the solar spectrum owing to the conversion of NIR light to UV or visible light. Therefore, constructing ZnIn_2_S_4_-based up-conversion materials is a promising approach to developing NIR-responsive photocatalysts. Among up-conversion materials, lanthanide (Ln) and carbon dots (CDs) are the most attractive to trigger NIR light-driven ZnIn_2_S_4_-based photocatalysts.

**Figure 3:**
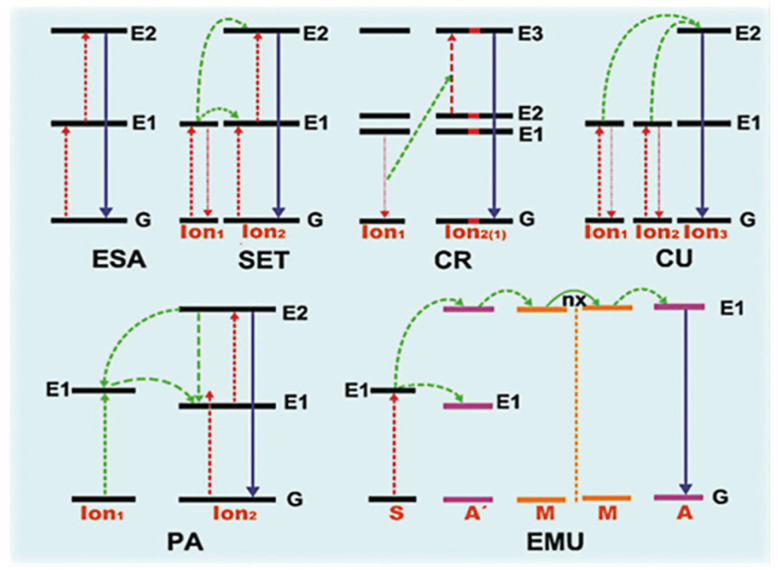
Principal diagram for the up-conversion processes of Ln^3+^–doped crystals, including excited-state absorption (ESA), successive energy transfer (SET), cross-relaxation (CR), cooperative up-conversion (CU), photon avalanche (PA), energy migration-mediated UC (EMU) mechanisms. The red, green, and purple lines stand for photon excitation, energy transfer, and emission processes, respectively. Reprinted with permission from Ref. [66]. Copyright 2021, John Wiley and Sons.

**Figure 4:**
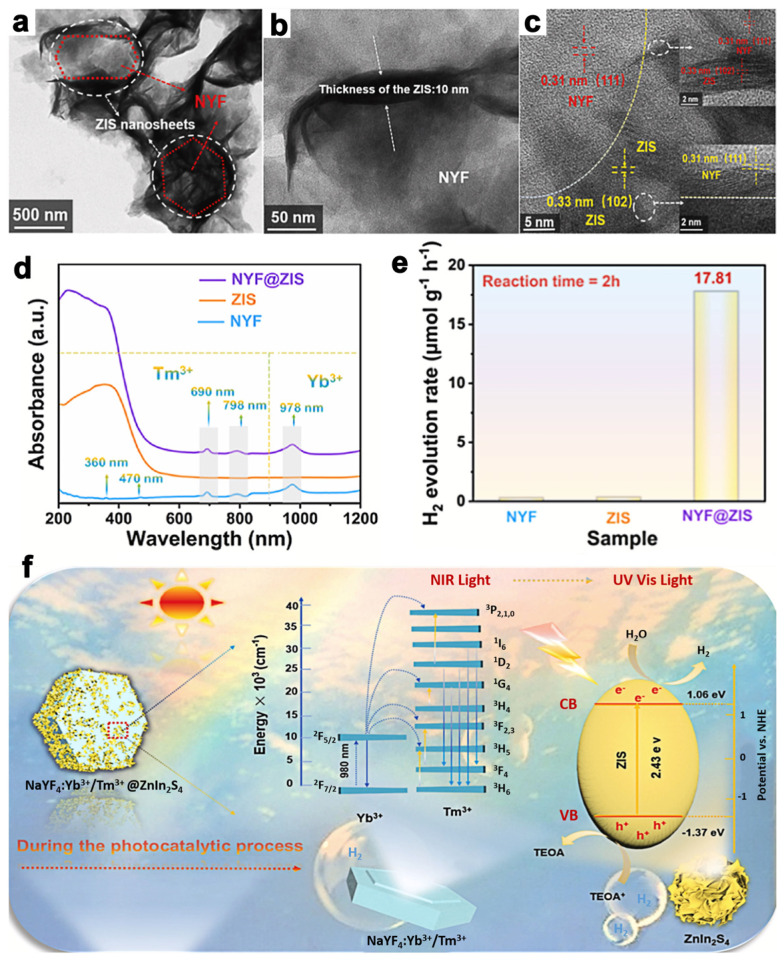
(**a**,**b**) TEM and (**c**) HRTEM images of NYF@ ZnIn_2_S_4_ composite. (**d**) UV–vis–NIR absorption spectra of ZnIn_2_S_4_, NYF and NYF@ ZnIn_2_S_4_ composite. (**e**) Time–dependent photocatalytic hydrogen production curves and hydrogen evolution rates of ZnIn_2_S_4_, NYF, and NYF@ZnIn_2_S_4_ composite under NIR (λ > 800 nm) light irradiation. (**f**) Mechanism of photocatalytic H_2_ evolution for NYF@ ZnIn_2_S_4_ materials under NIR light illumination. Reprinted with permission from Ref. [55]. Copyright 2022, Elsevier.

The up-conversion system utilizes the solar spectrum by the following mechanisms: excited-state absorption (ESA), photon avalanche (PA), energy migration-mediated UC (EMU), and energy transfer up-conversion (ETU) (Figure 3). Specifically, the ETU system includes successive energy transfer (SET), cross-relaxation (CR), and cooperative up-conversion (CU). Lanthanide (Ln)-based UC materials generally include two types of ions as the donor and acceptor, respectively; therefore the ion composition and matrix could be prominent factors for their efficiency. Host matrices such as NaYF_4_, NaGdF_4_, and CaF_2_ are commonly used, as well as ion compositions of Yb/Er, Yb/Tm, and Yb/Ho. Moreover, direct Ln ion doping and heterostructure development are common strategies to develop Lanthanide materials with ZnIn_2_S_4_. Shi and Guo et al. [55] have successfully prepared a three-dimensional NaYF: Yb^3+^/Tm^3+^@ZnIn_2_S_4_ (NYF@ZIS) material through a facile hydrothermal and water-bathing process, successfully leading to ZnIn_2_S_4_ coating onto the surface of hexagonal prisms (Figure 4a–f). NYF@ZIS exhibited intensive optical absorption ranging from 650 to 1000 nm, giving the property of utilizing NIR light. The UC process is ascribed to the energy transfer between Yb^3+^ and Tm^3+^ ions, and eventually, ZnIn_2_S_4_ was excited through the ET process from the up-conversion luminescence agent of NaYF:Yb^3+^/Tm^3+^. Furthermore, the separation of photogenerated charge carriers was promoted by the construction of ZnIn_2_S_4_ and NaYF:Yb^3+^/Tm^3+^. In this way, the application of H_2_ evolution for NaYF:Yb^3+^/Tm^3+^@ZnIn_2_S_4_ was tested under NIR light (λ > 800 nm) irradiation and showed an evolution rate of 17.81 µmol g^−1^ h^−1^. Coupling up-conversion nanoparticles (UCNPs, NaYF:Yb/Tm) enhanced the photocatalytic performance of ZnIn_2_S_4_ according to Li and Idris et al. [58], in which UCNPs absorbed NIR photons and emitted UV–visible photons to excite ZnIn_2_S_4_. Ultimately, the photoinduced energy transfer gave rise to remarkable CO and CH_4_ production rates of 1500 and 220 nmol g^−1^ h^−1^ under NIR-light illumination. Overall, the Ln doping promoted ZnIn_2_S_4_ photocatalytic performance by not only increasing available high-energy photons with the absorption range through up-conversion luminescence approaches but also by morphology modification for efficient separation and migration of photoinduced charge carriers.

**Figure 5:**
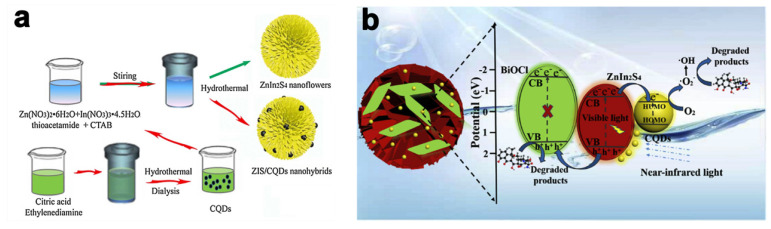
(**a**) Schematic illustration for the preparation method of ZnIn_2_S_4_/CQDs composites. Reprinted with permission from Ref. [59]. Copyright 2018, Elsevier. (**b**) Photocatalytic reaction mechanism of CQDs/ZnIn_2_S_4_/BiOCl heterostructures for antibiotic removal at visible and NIR region. Reprinted with permission from Ref. [60]. Copyright 2019, Elsevier.

Besides Ln-based ZnIn_2_S_4_ construction, carbon dots (CDs) are another up-conversion material for efficient sunlight utilization, which possesses properties of low toxicity, up- and down-conversion fluorescent, good electron transfer, and excellent photostability [67,68,69,70]. Xu et al. [59] constructed carbon quantum dots doped ZnIn_2_S_4_ nanoflowers (ZIS/CQDs) with a wide light response range through a simple in situ hydrothermal synthesis approach (Figure 5a). It demonstrated remarkably expanded absorption attributed to its up-converted mechanism, and the suppression of photoexcited charge recombination owing to the CQDs’ performance of photon harvest and transportation. Additionally, the doping content of CQDs also affected the photocatalytic removal efficiency of tetracycline hydrochloride (TCH), and the highest degradation rate reached almost three times higher than pure ZnIn_2_S_4_ irradiated under NIR light.

CQDs promoted the photocatalytic degradation performance of CQDs/ZnIn_2_S_4_/BiOCl for antibiotic removal at visible and NIR regions, as reported by Lu et al. [60], in which CQDs modified 2D BiOCl nanosheets/2D layered flower-like ZnIn_2_S_4_ with van der Waals (VDW) force in between. The as-prepared CQDs/ZnIn_2_S_4_/BiOCl heterostructures exhibited noteworthy degradation rates that were 42.5, 2.75, and 1.94 more times efficient than those of pristine BiOCl, ZnIn_2_S_4_, and ZnIn_2_S_4_/BiOCl photocatalysts, respectively. For the reaction mechanisms in CQDs/ZnIn_2_S_4_/BiOCl, CQDs absorbed NIR photons and emitted visible light photons to excite ZnIn_2_S_4_, resulting in electron migration from the valence band to the conduction band. On the one hand, the electrons migrated to CQDs reacting with oxygen in the water to produce reactive •O_2_^−^ and finally reacted with antibiotic compounds. On the other hand, holes in VB of ZnIn_2_S_4_ transferred to BiOCl and degraded pollutants (Figure 5b). The enhanced photocatalytic performance was attributed to the van der Waals forces between ZnIn_2_S_4_ and BiOCl, as well as the introduction of CQDs, which acted as electron acceptors, extended the light absorption edge to NIR and finally boosted photoinduced charge migration due to its up-conversion luminescence ability. Additionally, several approaches including electron spin-resonance spectroscopy (ESR), active species trapping experiments, and high-performance liquid chromatography–tandem mass spectrometry (LC–MS/MS), were carried out to elucidate the photocatalytic degradation pathway of antibiotics (TC, CIP, and OTC) and revealed that the reactive species (•O_2_^−^, OH, and holes) played important roles in the degradation processes. 

In general, although the up-conversion strategy is a promising way to promote ZnIn_2_S_4_ photocatalytic efficiency, it requires further modification for practical application. Based on the mechanism of up-conversion, improving the light overlap of Ln-based materials or carbon quantum dots emitted and ZnIn_2_S_4_ absorbed promotes the enhancement of solar energy utilization. Therefore, up-conversion materials with wide light absorption and photostability are needed for higher photocatalytic efficiency.

## 5. Surface Plasmon Resonance

**Figure 6:**
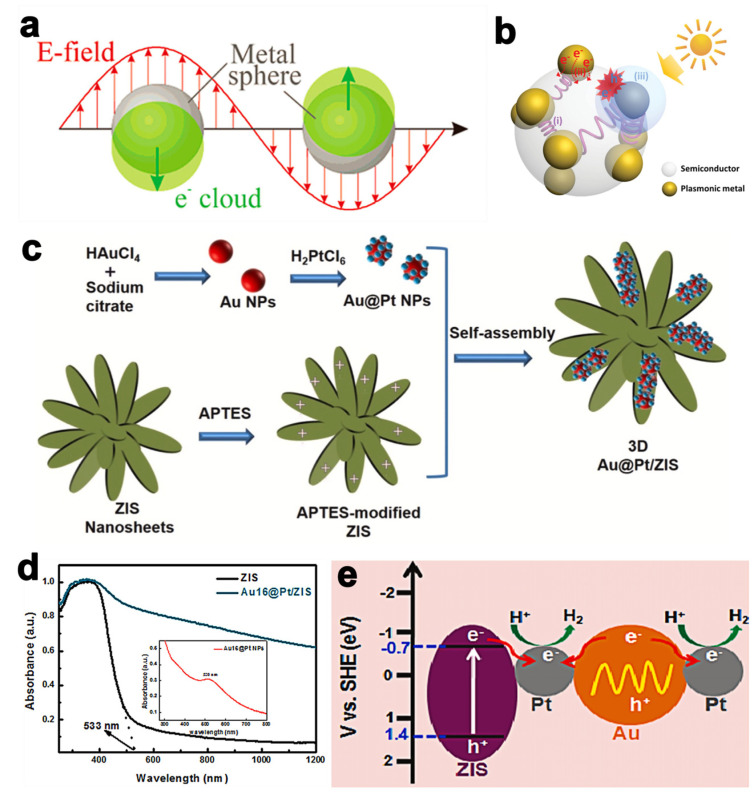
(**a**) Schematic illustration of plasmon oscillation on a plasmonic metal sphere [71]. (**b**) Mechanisms for photonic promotion in plasmonic nanoparticles–semiconductor heterostructures, including light scattering and concentration (i), direct electron injection energy transfer (DET) (ii), and plasmon–induced energy transfer (PIRET) (iii). Reprinted with permission from Ref. [71]. Copyright 2015, John Wiley and Sons. (**c**) Schematic illustration of the Au@Pt/ZnIn_2_S_4_ composites preparation. (**d**) UV–vis–NIR spectra of ZnIn_2_S_4_ and Au@Pt/ZnIn_2_S_4_. Inset is the absorption spectrum of colloidal Au@Pt nanoparticles. (**e**) Proposed excitation scheme for the hydrogen production of Au@Pt/ZnIn_2_S_4_ composites. Reprinted with permission from Ref. [63]. Copyright 2021, Elsevier.

Surface plasmon resonances (SPR) or localized surface plasmon resonances (LSPR) is defined as the electron coherent oscillations in metal nanoparticles in response to visible–NIR light irradiation (Figure 6a) [71,72]. On the one hand, the SPR effect triggered by plasmonic metals promotes the photocatalytic ability by absorbing a broader light range, and therefore, the process is affected by the size, shape, and dielectric environment of nanoparticles. Both light scattering and light concentration are essential to the absorption edge extension of photocatalysts by increasing the optical path owing to light trapping or photonic effects (Figure 6b) [72]. On the other hand, direct electron injection energy transfer (DET) and plasmon-induced energy transfer (PIRET) systems are constructed by integrating nanoparticles with semiconductors’ heterostructure to extend the light-response region, as well as accelerating charge carrier transfer in between (Figure 6b) [71,73]. Noble nanoparticles, such as Au, Ag, and Pt, are commonly introduced to form heterostructures resulting in the enhancement of ZnIn_2_S_4_ photocatalytic activity. Yin et al. fabricated assembling core–shell Au@Pt nanoparticles on 3D ZnIn_2_S_4_ microsphere composites by following multiple steps, first by using citrate reduction and solvothermal methods to form Au nanoparticles and ZnIn_2_S_4_, respectively, and obtain Au@Pt nanoparticles via electrostatic interaction force and eventually collect composites attributed to the electrostatic force and assist the precursors (Figure 6c) [63]. As shown in Figure 6c, the absorption edge of Au@Pt/ZnIn_2_S_4_ has been extended from the visible to the NIR light region compared with pure ZnIn_2_S_4_ and Au@Pt since the Au@Pt serves as the secondary light source by concentrating photons to ZnIn_2_S_4_, thus generating an EM field with higher energy than therefore the process is affected by the size, shape, and dielectric environment of nanoparticles surrounding light [74,75,76], which further promotes the utilization of light spectrum. Moreover, the electron hole separation efficiency is accelerated because electrons from both ZnIn_2_S_4_ and Au transfer to Pt nanoparticles according to the energy-band scheme (Figure 6d,e). Benefiting from these mechanisms above, Au@Pt/ZnIn_2_S_4_ experiences 10 times greater hydrogen evolution efficiency compared with pure ZnIn_2_S_4_ under vis–NIR light irradiation.

**Figure 7:**
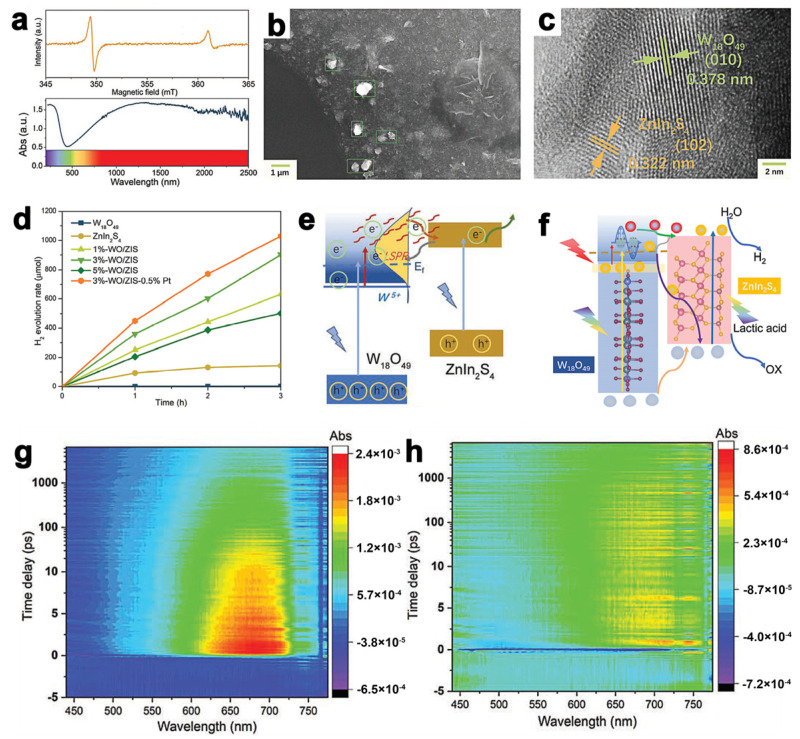
(**a**) Absorption spectra of W_18_O_49_. (**b**) SEM and (**c**) HRTEM images of W_18_O_49_/ZnIn_2_S_4_. (**d**) Time-dependent photocatalytic H_2_ evolution curves of different samples under simulated sunlight irradiation. (**e**) Schematic diagram of electron transition of W_18_O_49_/ZnIn_2_S_4_ under 400 nm irradiation. (**f**) The schematic diagram for photocatalytic H_2_ evolution by W_18_O_49_/ZnIn_2_S_4_ heterojunction. Pseudocolor TA plots of (**g**) ZnIn_2_S_4_ and (**h**) W_18_O_49_/ZnIn_2_S_4_ under 400 nm irradiation. Reprinted with permission from Ref. [62]. Copyright 2022, John Wiley and Sons.

In addition to noble nanoparticles, nonmetallic semiconductors show a plasmonic effect owing to the properties of element doping or lattice vacancies such as Cu_2-x_S, MoO_3-x_, WO_3-X,_ and W_18_O_49_ [77,78,79,80,81]. Among them, W_18_O_19_ has strong absorption in the visible–NIR wavelengths (Figure 7a) and thus is a promising candidate for developing ZnIn_2_S_4_ NIR hybrid photocatalysts as a result of sufficient vacancy on its surface [62]. For example, Zhang et al. [62] prepared the W_18_O_49_/ZnIn_2_S_4_ composites (Figure 7b,c) with a full-spectrum sunlight response with excellent hydrogen evolution under UV and NIR light illumination (Figure 7d). The mechanisms of W_18_O_49_/ZnIn_2_S_4_ for hydrogen evolution were proposed as shown in Figure 7e,f. The DFT calculation is conducted to prove that W^5+^-W^5+^ pairs are the prerequisite for the LSPR effect of W_18_O_49_. Multiple additional approaches, such as ultrafast transient absorption spectroscopy (TA) and ESR, are applied to investigate the mechanisms, and the results show that W_18_O_49_ captures two photons to excite ZnIn_2_S_4_, which provides more opportunity for ZnIn_2_S_4_ to utilize solar spectrum. Moreover, as in the ultrafast transient absorption spectroscopy (TA) measurement, in the plots of pure ZnIn_2_S_4_ (Figure 7g) and W_18_O_49_/ZnIn_2_S_4_ (Figure 7h) with the pump light at 400 nm, the ZnIn_2_S_4_ image exhibits a higher concentration of short-lived electrons than that of W_18_O_49_/ZnIn_2_S_4_ at the signal range from 550 to 750 nm, which indicates that more photoinduced electrons recombine with holes in the ZnIn_2_S_4_ than W_18_O_49_/ZnIn_2_S_4_. In this way, it is concluded that in the W_18_O_49_/ZnIn_2_S_4_ heterostructure, the recombination of electrons and holes in ZnIn_2_S_4_ is suppressed, and thus, the transfer efficiency of photoexcited charge carriers is accelerated due to the Z-scheme construction of W_18_O_49_ and ZnIn_2_S_4_. 

**Figure 8:**
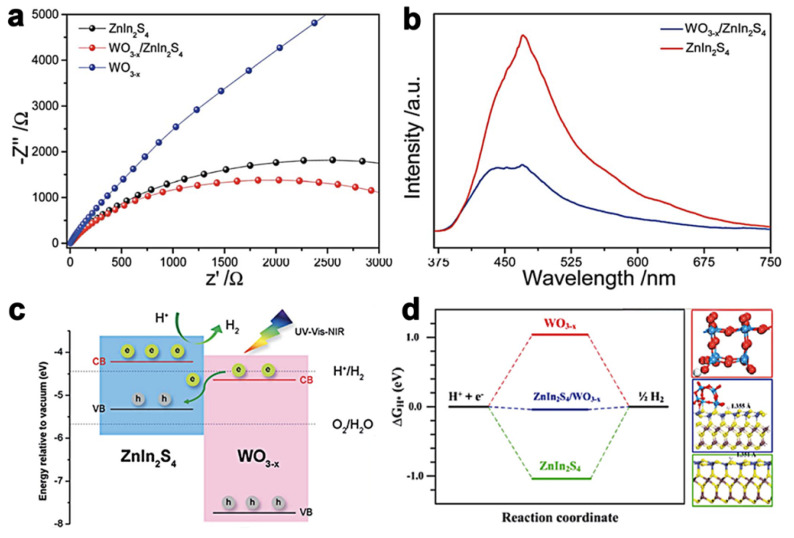
(**a**) Electrochemical impedance spectroscopy (EIS). (**b**) Photoluminescence and (**c**) diagram of the proposed H_2_ production mechanism for WO_3-X_/ZnIn_2_S_4_. (**d**) DFT calculation △G_H*_ values and adsorption property of ZnIn_2_S_4_, WO_3-X_ and WO_3-X_/ZnIn_2_S_4_. Reprinted with permission from Ref. [56]. Copyright 2021, Royal Society of Chemistry.

Apart from W_18_O_49_, WO_3-X_ with lattice vacancy also has a surface plasmon resonance (SPR) effect owing to its collective oscillations of free carriers. Ni et al. [56] fabricated WO_3-X_/ZnIn_2_S_4_ heterojunctions via a straightforward scaled-up process for H_2_ production photoactivity. The interaction of WO_3-X_ with ZnIn_2_S_4_ is beneficial for expanding the absorption light from UV–visible to NIR light range, which gives the photocatalysts capability to generate H_2_ by splitting water throughout the full solar spectrum. WO_3-X_/ZnIn_2_S_4_ photocatalysts exhibit a remarkable H_2_ production rate of 20,957 µmol h^−1^ g^−1^, which is 2.7 times higher than that of pure ZnIn_2_S_4_ under simulated sunlight illumination. Together with the results of the EIS (Figure 8a) and PL measurements (Figure 8b), the WO_3-X_/ZnIn_2_S_4_—with a smaller resistance curve and weaker PL emission than pure ZnIn_2_S_4_—indicates more efficient electron transfer and charge separation. Additionally, the proposed mechanism (Figure 8c) reveals that after absorbing UV–vis–NIR light, electrons in the VB of WO_3-X_ and ZnIn_2_S_4_ migrate to their respective CB, then electrons and holes in the VB of ZnIn_2_S_4_ combine together as a result of the electron transfer from CB of WO_3-X_. The H_2_ production eventually occurs in the CB of ZnIn_2_S_4_. The △G_H*_ value of the WO_3-X_/ZnIn_2_S_4_ hybrid calculated by the DFT method further confirms the mechanism (Figure 8d).

**Figure 9:**
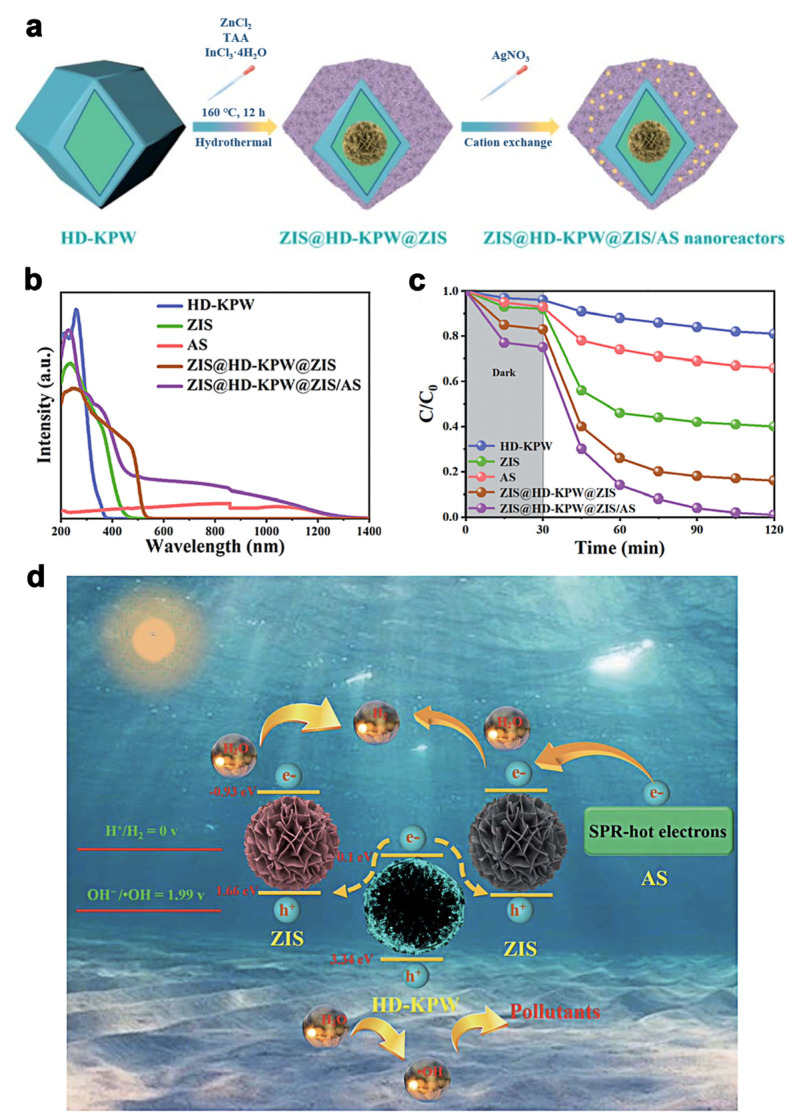
(**a**) Schematic illustration of K_3_PW_12_O_40_@ZnIn_2_S_4_/Ag_2_S preparation, hollow dodecahedral K_3_PW_12_O_40_, ZnIn_2_S_4_ and Ag_2_S were named HD-KPW, ZIS and AS. (**b**) UV–Vis–NIR diffuse reflectance spectra and (**c**) tetracycline hydrochloride photodegradation of K_3_PW_12_O_4_, ZnIn_2_S_4_, Ag_2_S, K_3_PW_12_O_40_@ZnIn_2_S_4_, and K_3_PW_12_O_40_@ZnIn_2_S_4_/Ag_2_S, respectively. (**d**) Proposed mechanism of K_3_PW_12_O_40_@ZnIn_2_S_4_/Ag_2_S heterojunctions for pollutant removal and hydrogen production. Reprinted with permission from Ref. [61]. Copyright 2022, Royal Society of Chemistry.

In another study, Zhou et al. [61] synthesized a novel photocatalyst of K_3_PW_12_O_40_@ZnIn_2_S_4_ and further loaded Ag_2_S quantum dots via the hydrothermal method followed by a cation exchange process, construction a dodecahedral K_3_PW_12_O_40_ capped with flower-like ZnIn_2_S_4_, and finally by distribution of Ag_2_S dots on the surface (Figure 9a). The introduction of Ag_2_S quantum dots led to a red shift from visible light to NIR light with higher optical absorption intensity (Figure 9b) compared with pristine ZnIn_2_S_4_ and K_3_PW_12_, respectively. It displayed high photocatalytic performance and photostability both for tetracycline hydrochloride and H_2_ production activity, notably with an optimal photodegradation rate of 99% (Figure 9c) and hydrogen production rate of 2.1 mmol g^−1^ h^−1^. Overall, the boosted NIR photoactivity was attributed to the SPR effect of Ag_2_S quantum dots (Figure 9d), which have extensive absorption of NIR light. Furthermore, the unique structure of K_3_PW_12_O_40_@ZnIn_2_S_4_/Ag_2_S provided more efficient light scattering and reactive reaction sites and prolonged the lifetime of photoinduced charge carriers.

## 6. Conclusions and Prospects

In recent years, ZnIn_2_S_4_ has come to be regarded as a promising photocatalyst due to its excellent properties of low cost, facile synthesis, and environmental harmlessness. Benefiting from these features, it has been widely applied in organic pollutant removal, hydrogen evolution, CO_2_ reduction, etc. Nevertheless, its photocatalytic efficiency is far from satisfactory due to its limited NIR light absorption, and thus, the fabrication of ZnIn_2_S_4_-based NIR photocatalysts is highly desired to enhance NIR light utilization. The present review summarizes strategies for promoting the extension of ZnIn_2_S_4_ response into the NIR range, as well as an overview of their fabrication methods, mechanisms for reactions, and applications. Strategies including hybrid with narrow optical gap materials, bandgap engineering, up-conversion materials, and surface plasmon resonance are overviewed to extend ZnIn_2_S_4_’s NIR light absorption and boost its solar energy efficiency. In addition, the photocatalytic applications of NIR-driven ZnIn_2_S_4_-based photocatalysts have been reported in the field of hydrogen production, pollutant removal, and CO_2_ reduction. 

Despite the promising results that have been accomplished, there remain several primary challenges to overcome. (1) The NIR absorption range of ZnIn_2_S_4_-based photocatalytic systems should be further expanded to make better utilization of solar energy. More research on constructing ZnIn_2_S_4_ with up-conversion or SPR materials is needed to enhance the absorption range. (2) Photosensitizers can absorb NIR photons to generate electrons, and if combined with ZnIn_2_S_4_-based photocatalysts, could further promote the NIR utilization of the ZnIn_2_S_4_–hybrid photosystems. However, no fabrications and applications of photosensitizers/ZnIn_2_S_4_ composites have yet been reported. More efforts could be devoted to this photocatalysis direction. (3) The light absorption intensity of the NIR ZnIn_2_S_4_-based photocatalysts is relatively inadequate, which results in low efficiency from solar to chemical energy. Therefore, modifications to the structure, morphology, and composition of SPR materials should be explored, which could enhance the NIR intensity by concentrating light and extending the absorption edge. (4) The mechanism of ZnIn_2_S_4_-based photosystems for NIR absorption is unclear; further approaches such as DFT calculations and advanced technics are strongly recommended to investigate the NIR absorption principles. (5) Future lab-scale and practical research on constructing easy-synthesis and environmentally friendly NIR ZnIn_2_S_4_-based photocatalysts is required with the aim to apply the photocatalysis technology to industrial applications.

## Figures and Tables

**Table 1:** Summary of NIR-driven ZnIn_2_S_4_-based NIR photocatalysts for various applications.

Photocatalysts	Broadband Light Harvester	NIR Photon Capture Method	Light Source	Extended Wavelength	Application	Ref.
ZIS/AgIn_5_S_8_	AgIn_5_S_8_	Hybrid with NOGMs	500 W tungsten halogen lamp	>420 nm	Dye degradation	[57]
ZIS/CuInS_2_	CuInS_2_	Hybrid with NOGMs	300 W Xe-lamp	>420 nm	H_2_ production	[53]
Zn-defective ZIS-Laponite	Laponite	BGE	Visible light	400–800 nm	Dye degradation	[54]
NaYF_4_:Yb,Tm/ZIS	NaYF_4_:Yb,Tm	Up-conversion effect	300 W Xe-lamp	≥800 nm	CO_2_ reduction	[58]
NaYF_4_:Yb^3+^/Tm^3+^@ZIS	NaYF_4_:Yb^3+^/Tm^3+^	Up-conversion effect	300 W Xe-lamp	<400 nm 400–800 nm >800 nm	H_2_ production	[55]
CQDs/ZIS	CQDs	Up-conversion effect	150 W infrared lamp	N.A.	Tetracycline hydrochloride degradation	[59]
CQDs ZIS/BiOCl	CQDs	Up-conversion effect	300 W Xe-lamp 150 W infrared lamp	>420 nm <700 nm	Antibiotics removal	[60]
WO_3-x_/ZIS	WO_3-x_	SPR	300 W Xe lamp	400–1100 nm	H_2_ production	[56]
K_3_PW_12_O_40_@ZIS/Ag_2_S	Ag_2_S	SPR	300 W Xe-lamp	>420 nm	H_2_ production Tetracycline hydrochloride degradation	[61]
W^5+^–W^5+^ pair induced of W_18_O_49_/ZIS	W_18_O_49_	SPR	Simulated solar light NIR light	>420 nm >700 nm	H_2_ production	[62]
Au@Pt/ZIS	Au@Pt	SPR	300 W Xe-lamp	≥420 nm	H_2_ production	[63]
ZIS/N-doped graphene	N-doped graphene	Photothermal effect	300 W Xe-lamp	>420 nm	CO_2_ capture CO_2_ photoreduction	[33]
SnSe/ZIS	SnSe	Photothermal effect	300 W Xe lamp	400–1100 nm	H_2_ production	[50]

ZIS: ZnIn_2_S_4_; NOGMs: narrow optical gap materials; BGE: bandgap engineering; SPR: surface plasmon resonance; CQDs: carbon quantum dots.
